# Frequency, Magnitude, and Possible Causes of Stranding and Mass-Mortality Events of the Beach Clam *Tivela mactroides* (Bivalvia: Veneridae)

**DOI:** 10.1371/journal.pone.0146323

**Published:** 2016-01-08

**Authors:** Alexander Turra, Maíra Pombo, Marcelo Petracco, Eduardo Siegle, Mariana Fonseca, Márcia R. Denadai

**Affiliations:** 1 Instituto Oceanográfico, Universidade de São Paulo, São Paulo, São Paulo, Brazil; 2 Faculdade de Oceanografia, Instituto de Geociências, Pós-Graduação em Ecologia, Universidade Federal do Pará, Belém, Pará, Brazil; 3 Centro Universitário Módulo, Caraguatatuba, São Paulo, Brazil; Universidade Federal do Rio de Janeiro, BRAZIL

## Abstract

Stranding combined with mass-mortality events of sandy-beach organisms is a frequent but little-understood phenomenon, which is generally studied based on discrete episodes. The frequency, magnitude, and possible causes of stranding and mass-mortality events of the trigonal clam *Tivela mactroides* were assessed based on censuses of stranded individuals, every four days from September 2007 through December 2008, in Caraguatatuba Bay, southeastern Brazil. Stranded clams were classified as dying (closed valves did not open when forced) or dead (closed valves were easily opened). Information on wave parameters and the living intertidal clam population was used to assess possible causes of stranding. This fine-scale monitoring showed that stranding occurred widely along the shore and year-round, with peaks interspersed with periods of low or no mortality. Dead clams showed higher mean density than dying individuals, but a lower mean shell length, attributed to a higher tolerance to desiccation of larger individuals. Wave height had a significant negative relationship to the density of dying individuals, presumed to be due to the accretive nature of low-energy waves: when digging out, clams would be more prone to be carried upward and unable to return; while larger waves, breaking farther from the beach and with a stronger backwash, would prevent stranding in the uppermost areas. This ecological finding highlights the need for refined temporal studies on mortality events, in order to understand them more clearly. Last, the similar size structure of stranded clams and the living population indicated that the stranded individuals are from the intertidal or shallow subtidal zone, and reinforces the ecological and behavioral components of this process, which have important ecological and socioeconomic implications for the management of this population.

## Introduction

Stranding and mass mortality on the shore are common events for many invertebrate groups, and their frequency, extent, and duration have been increasingly reported in recent decades [1–3]. These events are attributed to several factors, such as storms, positive sea-temperature anomalies, parasitism, harmful algal blooms, and hypoxia [1,2,4–12]. Despite the ecological importance of these processes, data on the frequency and magnitude of stranding and mass-mortality events of invertebrates are generally limited in time, probably because of the sudden and generally episodic occurrence of this phenomenon [2,3,13], for sandy-beach clams in particular [2,9,10,13–17]. Mass mortality of sandy-beach clams has important ecological implications because it can affect the density of these frequently dominant populations, altering the structure of the benthic community [18,19], as well as the organisms that rely on this food source [20]. In extreme cases, mass mortality can decimate clam populations along their geographical range, as reported for the yellow clam *Mesodesma mactroides* and the surf clam *M*. *donacium* [9,11,21–23]. In addition, in view of the high importance of sandy-beach clams in recreational and artisanal fisheries of developing countries in South America, mass-mortality events of these organisms can be socioeconomically significant [18,22,24–26]. The trigonal clam *Tivela mactroides* is an important sandy-beach species, both economically and ecologically [20,27–34]. It has a wide distribution from Venezuela to Brazil (Pará to Santa Catarina states) and in the West Indies [35]. This suspension-feeder inhabits the intertidal and subtidal zones of sandy beaches, where it is often the dominant macrofaunal component [28,36–38]. The trigonal clam frequently reaches high abundance in Venezuelan beaches, where it is an important resource of artisanal fisheries [28,30,31]. On these tropical beaches, no studies of mass mortality of the trigonal clam have been conducted, although there are brief notes on observations made under conditions of strong winds and waves [28]. The population of the trigonal clam living in the intertidal zone of sandy beaches in Caraguatatuba Bay, southeastern Brazil, has continuous recruitment, high individual growth and mortality rates, and reaches high levels of density, biomass and secondary production [32,33]. On several occasions, large numbers of stranded clams were observed in the intertidal zone of these beaches, and these mass strandings were presumed to be related to population density-dependent processes such as individual growth and mortality rates [32]. Also for this population, an assessment of harvesting intensity showed that under ordinary conditions, the amount of clams harvested yearly is about 25 tons. The same study showed that the mass-mortality events are also socioeconomically important, since a decrease in harvesting was attributed partly to these events [34]. The authors suggested that the foul odor and unpleasant sight caused by the stranded and dead clams were responsible for a decrease in recreational use of the beaches in Caraguatatuba Bay. Mass-mortality events can have dramatic effects on fishing and management of natural resources [11,22]. Moreover, some physical changes related to global climate change can increase the frequency and severity of mass-mortality episodes on sandy beaches [9,11]. In view of these considerations, assessment of mortality events of *T*. *mactroides* is a crucial step in managing populations of this species.

Among the many possible causes of mortality are aspects of the population dynamics such as overpopulation, competition for resources, and parasitism, but also physical features, since these clams live under harsh conditions, depending largely on the characteristics of the sediment where they burrow and on the wave energy across the shore [9,11,17,32]. Wave energy has a high potential to transport sediment as well as individual clams, while the sediment conditions may favor or preclude their burrowing and survival [39]. In fact, shoreward passive migration of small-sized clams was hypothesized to explain spatial partitioning between small (subtidal) and large (intertidal and shallow subtidal) individuals [37].

The present study aimed to shed some light on stranding and mass-mortality events of these beach clams, by assessing their frequency, magnitude and possible causes for the population of *T*. *mactroides* in Caraguatatuba Bay. A comprehensive study was carried out on stranded individuals, both dying and dead, at a 4-day temporal scale during 15 months. The concomitant physical and biological conditions were assessed in relation to the stranding events. Given the general importance of hydrodynamics for mortality events on sandy beaches [40], wave parameters were considered in order to test the hypothesis that these are related to the number or size of stranded individuals. Similarly, the data for intertidal-living buried individuals, obtained by Turra et al. [32], were also included as a potential factor influencing the structure of stranded individuals, i.e., if the density or size of the stranded clams is related to the respective parameters of the buried intertidal individuals.

## Material and Methods

### Study area and sampling

The coastline of Caraguatatuba Bay (23°37’S to 23°44’S and 45°24’W to 45°26’W) in southeastern Brazil is about 16 km long and is uninterrupted by physical barriers. The sandy beaches in the study area have a gentle slope (1/33-1/41 m), a wide surf zone (*ca* 150 m), fine to very fine sand (0.8–1.12 mm), and waves predominantly with a period of 6 s and height of 0.4 m [32,33,37].

**Fig 1:**
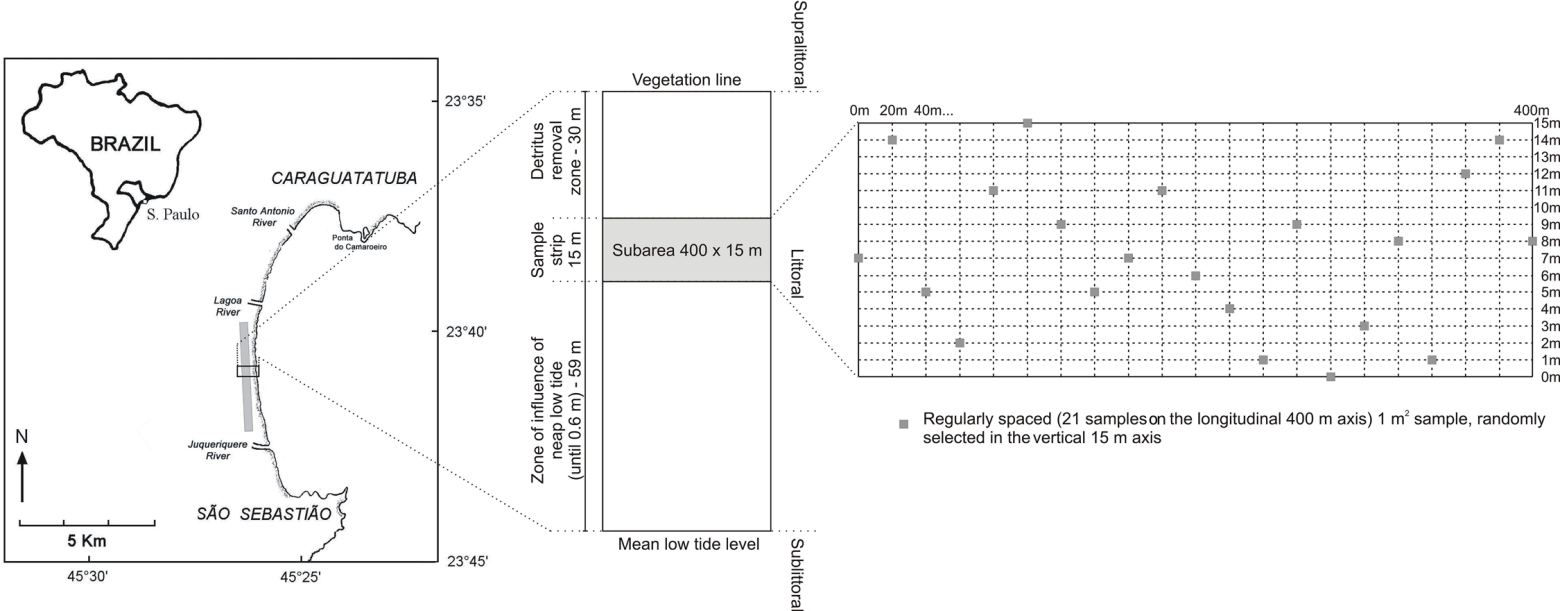
Study area and sampling scheme. Left: map of Caraguatatuba Bay; in gray, the 4-km stretch where the sampling was conducted. Samples were taken every 4 days, in three randomly selected subareas (black rectangle). Center: plan of one sampling subarea, a strip 400 m long (parallel to the waterline) and 15 m wide (perpendicular to the waterline). The 15-m strip was established from the edge of neap low tide influence up to 30 m below the vegetation edge, to include recently stranded clams. Right: plan of one sampling strip, which was gridded every 20 m along shore, totaling 21 sampling stations. Across shore, one of 15 possibilities (0–15 m) was randomly selected for each sampling station. The 21 sampling units in each subarea were examined using a 1 m^2^ quadrat.

The mortality of *Tivela mactroides* was assessed in a 4-km area of the central-southern portion of the bay. This area was selected because intertidal densities of this clam are higher in this part of the bay [32,37]. Sampling was carried out under license granted by the *Instituto Brasileiro do Meio Ambiente e dos Recursos Naturais Renováveis* (IBAMA-DIREN No. 08/2001), at four-day intervals from September 8, 2007 to December 3, 2008. Preliminary evaluations indicated that this periodicity was sufficient to include all mortality events in the samples. Individuals stranded on the beaches were sampled in three subareas, each 400 m long (parallel to the waterline) and 15 m wide (perpendicular to the waterline), which were randomly selected on each sampling day in the study area (Fig 1). This 15-m strip was established 30 m below the vegetation edge (supralittoral limit), which was not included in the sampling, in order to avoid the effect of debris removal by the local government. Also, the area below the 15-m strip, which corresponded to the lower intertidal zone, was not included in the sampling design due to the impossibility of sampling during neap low tides. In contrast to the higher levels, where the stranded individuals generally had open valves, in this 15-m strip most of the stranded clams were found with the valves closed, indicating that they reached the beach strip recently.

In each subarea of 400 x 15 m, a rectangular grid was then defined, in which a sampling station was systematically established every 20 m (0, 20, 40, 60, … 400 m) along the longitudinal axis of the subarea, totaling 21 sampling stations (Fig 1). Across the shore, a number among 15 possibilities (0, 1, 2, 3,…15 m) was randomly selected for each sampling station. The 21 sampling units in each subarea were examined using a 1 m^2^ quadrat. In each quadrat, all stranded individuals of *T*. *mactroides* were sampled and separated into dying (closed valves that did not open when forced) or dead (open valves or easily opened when forced). The clams were placed in labeled plastic bags, and subsequently counted and measured for total length (in mm).

### Wave modeling

The height, period and power of the local waves were estimated daily for the entire sampling period (September 1, 2007 to December 3, 2008). Based on the regional offshore wave information extracted from the global wave generation model WaveWatch III (NCEP/NOAA) [41], a numerical model was applied in order to propagate the waves onshore until they reached the area of interest. The numerical model was the Delft3D (open-source version from Deltares) WAVE module, which simulates the propagation and transformation of wind-generated waves as they move over varying bottom morphology [42]. It simulates effects such as wave refraction, diffraction, shoaling and set-up in coastal areas.

The Caraguatatuba model used in this application covered most of the northern coast and continental shelf of São Paulo State, keeping the area of interest well inside the model domain. Grid resolution varied in the domain, with increased resolution onshore. Boundary wave data, defined at the shelf break, represented the wave climate (significant wave height and corresponding wave period) for the entire period of analysis, with information given at six-hour intervals. Subsequently, near-shore wave data were obtained at the entrance of Caraguatatuba Bay (23°40’S and 45°22’W), providing the local wave conditions during the sampling period. The daily mean wave height and period were calculated from four daily values. In order to account for the synergy of wave height and period, the wave power *P* was estimated by: *P = ρ g 2HT/32Π*, where *ρ* is water density (1,027 kg/m^3^), *g* the acceleration due to gravity (9.81 m/s^2^), *H* the wave height (m), and *T* the wave period (s). *P* is given in W/m.

### Data on living buried individuals

Considering that some population parameters (e.g., density and shell length) of the intertidal buried individuals can provide important information on the stranding process, the data for these clams obtained by Turra et al. [32] were also used. This study sampled buried individuals in summer and winter 2007 and 2008. Each sampling period included three random intervals of spring low tides, when living, buried clams were collected, counted and measured for total length (in mm).

### Data analysis

#### General characterization

The mean density (ind.m^-2^; total number of occurrences divided by 21, which is the mean number of individuals per 1 m^2^ quadrat within the area) and mean shell size (mm) were calculated separately for dying and dead stranded individuals for each area sampled. The strength of the relationship between dying and dead clams was evaluated for both density and shell size (for all cases the ordinary least square regression was appropriate).

Another goal was to assess temporal differences, as well as if there were differences according to the individuals' condition (dying or dead), in mean density and size of stranded individuals. Therefore, for each biotic measurement (density and size), months and condition were considered as factors in a factorial ANOVA for mixed design, where data on dead and dying individuals within a sample were considered repeated measures. The assumptions were all assessed and met, except for density, which showed a wide range of values, and consequently a high heterogeneity of the variances. Therefore, the density data were transformed (log_10_(x+1)) to meet the ANOVA requirements [43].

#### Relationship to wave conditions and to the living population structure

To assess whether the wave regime was related to the stranding of *T*. *mactroides*, mean daily values for density and size of stranded individuals were modeled separately according to wave height, period and power. Because the population may show a delayed response to a change in environmental conditions, for the wave data we used the parameters from the sampling day (time 0) and from the preceding days, using a time lag from one (time -1) to four days (time -4). Because these wave data are highly correlated, exploratory analysis and nested model selection were applied to determine which parameters should be used in the model without the inclusion of correlated factors. Model selection was performed [44,45] using a quantile regression analysis, more precisely the upper limit [46,47]. This technique shows the extreme state of a relationship, here considered of key importance because other factors, for example the lack of living individuals, would result in values well below this limit, and not contribute to the true shape of the relationship between the variables [48].

This modeling process indicated that the use of dying clams alone responded better to the environmental factors, and therefore better than the total number of stranded clams. Therefore, data on dead individuals were not used in model analyses. Because the transects were placed randomly in each sampling event, and the total area was too large to be fully cleared between sampling events, dead clams could have been pseudoreplicated. However, although among the stranded individuals a dead clam could be present longer than 4 days, a dying clam had certainly stranded recently enough to prevent an overlap between sampling periods.

Optimum model selection was performed by applying backward stepwise selection based on Akaike criteria and using hypotheses tests for each model run. Graphical analysis of the residuals was used to check for homogeneity of variances, normality and independence, until an ideal model meeting all assumptions was met [44]. Here, because a clear non-linear relationship was found, a generalized additive model (GAM), with Gaussian distribution and using a regression spline (thin plate) as a smoothing basis, was applied using the mgcv R package [49].

Data from Turra et al. [32] on the structure of the intertidal stratum of the living population were also used to investigate a possible relationship of this part of the living population to the structure of the stranded individuals. These data were sparser in time than the present study, and therefore only the corresponding monthly values were isolated and used in the analysis, which included all stranded individuals. Similarly to the previous analysis, exploratory data assessments were performed to validate this choice, and as expected given the broader time scale, the use of both dying and dead individuals proved to be better. The shell length and density of living individuals, obtained from Turra et al. [32], were compared with the shell length and density of the present stranded individuals, and an ordinary least square regression was used to assess the relationship in both cases, followed by a paired *t*-test for shell length.

## Results

### General characterization

**Fig 2:**
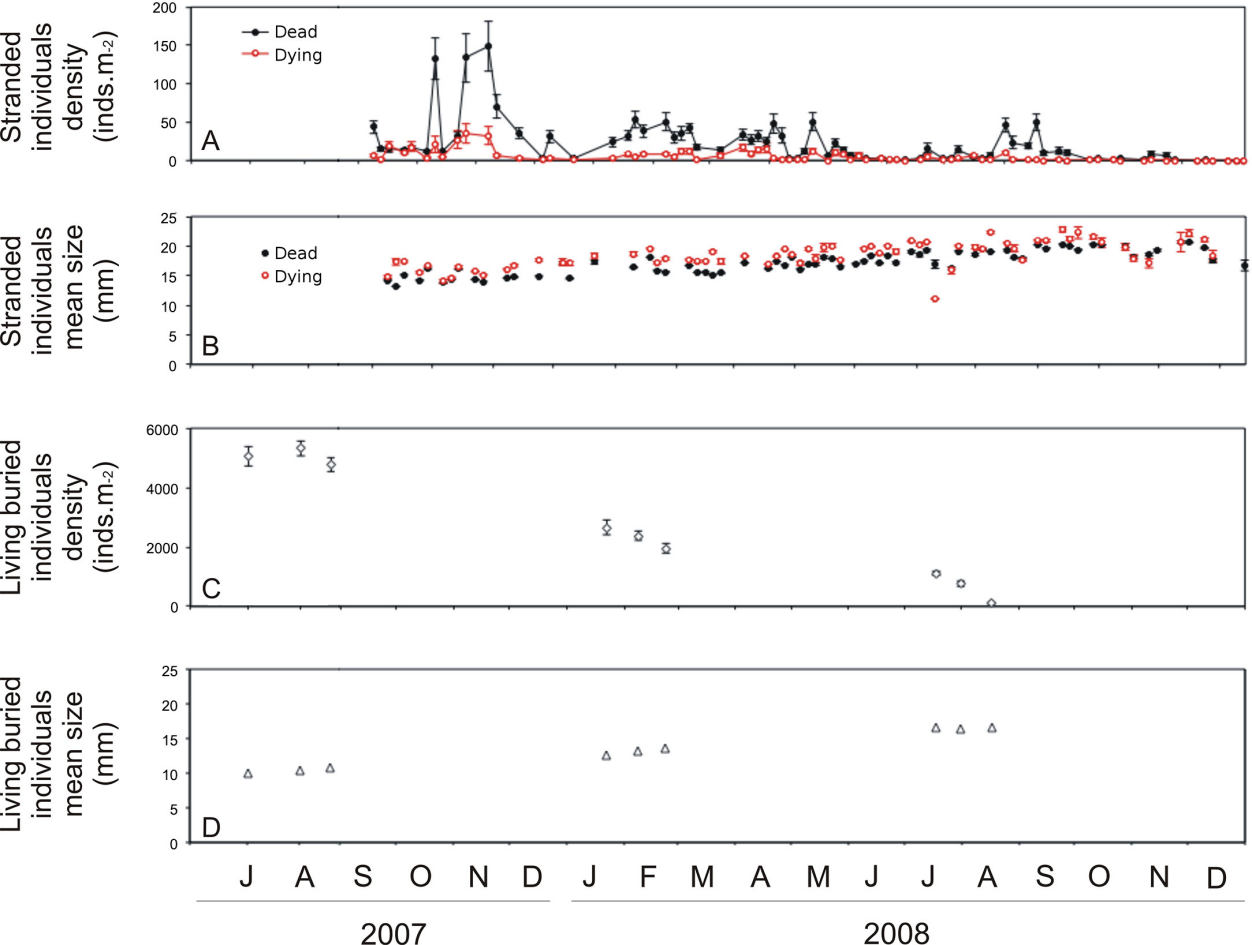
Temporal variation in *Tivela mactroides* stranded and living individuals. (a) Mean density ± SE (ind.m^-2^) and (b) mean length (mm) of stranded dying (red, hollow dots) and dead (black, full dots) individuals; (c) mean density ± SE (ind.m^-2^) and (d) mean length ± SE (mm) of intertidal buried individuals (the latter two from Turra et al. [32]).

A total of 118,871 stranded clams, of which 23,424 were dying (20%) and 95,447 were dead (80%), were collected in the 75 sampling events. Most of the quadrats and subareas sampled contained both dying and dead stranded individuals. Relationships between dying and dead clams were positive for both density (F_1,223_ = 253.5, p<0.01, r^2^ = 0.53) and shell length (F_1,166_ = 105.6, p<0.01, r^2^ = 0.39), showing that there was a significant, though weak, correspondence between dead and dying clams. The density of stranded clams differed significantly over time (F_15,32_ = 4.40, p<0.01), with an overall mean value of 27.09 ind.m^-2^ (± 45.87 SD) and wide oscillations throughout the study period (Fig 2A). Clam condition also differed significantly over time (F_1,32_ = 63.0, p<0.01), with the density of dying clams consistently lower (5.30 ± 10.15 ind.m^-2^) than that of dead ones (21.79 ± 38.03 ind.m^-2^). The abundances of dead and dying clams were positively correlated. However, although both conditions showed similar temporal patterns, for dying clams the differences were less variable, resulting in a significant interaction between factors (F_15,32_ = 4.45, p<0.01). This interaction, allied to the possibility of pseudoreplicating the estimates for dead clams, supported the decision to perform further modeling only for dying clams. Regarding the temporal pattern, high densities of stranded individuals were observed in October (80.35 ± 91.35 ind.m^-2^) and November 2007 (98.58 ± 134.13 ind.m^-2^), when about 35% of the total sampled stranded individuals were recorded. The density of stranded clams decreased after November 2007, remaining low but still oscillating, until reaching low values by the end of 2007. In 2008, high densities of stranded individuals were observed from January through June, with oscillating frequencies, and then decreased, with isolated peaks between August and October. In the last sampling events, stranded individuals were recorded only once.

Regarding mean shell length, dead clams were recurrently smaller then dying ones (F_1,26_ = 87.70, p<0.01; 17.06 ± 2.16 mm and 18.37 ± 2.46 mm, respectively) (Fig 2B). There was also a significant temporal difference in shell length (F_12,28_ = 37.41, p<0.01), with no interaction with individual condition (F_1,26_ = 0.75, p = 0.69). For both conditions, the values increased continuously over the sampling period (except for an outlier with small clams stranded in August 2008), from about 13 to 20 mm. Higher fluctuations in size were observed toward the end of the period, when densities were among the lowest.

### Relationship to waves

**Fig 3:**
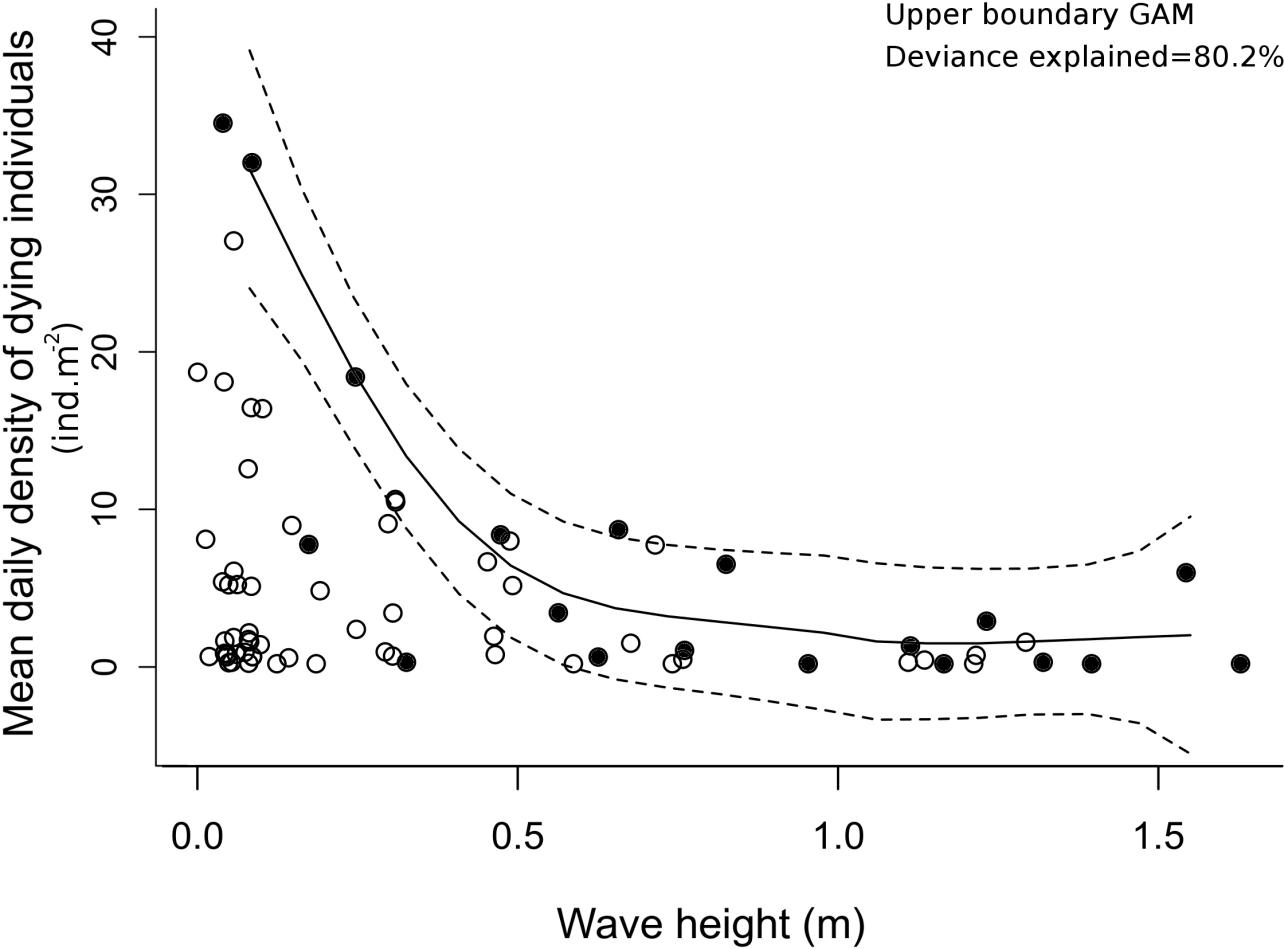
Relationship between density of stranded *Tivela mactroides* individuals and wave height. Density of dying stranded clams according to the significant explanatory variable, the wave height three days before the sampling. The upper boundary of the envelope was used (black dots, n = 19) to define the optimum model. Solid and dashed lines represent the optimum Generalized Additive Model and its 95% confidence bands respectively.

The density of dying clams showed negative relationships to wave height and power for all lag periods, from 0 to 4 days, and no apparent relationship to wave period for time lag. Indeed, based on the model selection process, the significant factor was the wave height on day lag -3, i.e., the wave heights three days before the samplings. Applying the upper boundary selection resulted in a decreasing exponential relationship between the number of dying individuals and increasing wave height (Fig 3). The selected Generalized Additive Model (GAM; F = 12.07, effective d.f. = 3.73, n = 19, p<0.001) explained 80.2% of the data deviance. The size of stranded individuals showed no relationship to any physical parameter (global F_6,162_ = 1.535, p = 0.170).

### Relationship to the living population structure

A sharp decrease of about 15 times in the density of the *T*. *mactroides* population was recorded by Turra et al. [32] between summer and winter 2007 (Fig 2C). The shell length increased continuously from late summer, until reaching its highest mean values in winter 2008, i.e., an inverse pattern to the density (Fig 2D). In the month just prior to the period when the stranded individuals were sampled (August 2007), the buried living individuals had a mean shell length of 13.0 mm; in August 2008, their mean length was 20.0 mm.

The size of stranded individuals showed a strong relationship to the size of the living population (F_1,5_ = 43.47, p<0.01, r^2^ = 0.90), and the difference in the mean lengths was not significant (paired *t* test; t_6_ = -1.74, p = 0.11). The density of stranded individuals, in turn, showed no relationship to the population density (F_1,5_ = 0.01, p = 0.92).

## Discussion

The fine-scale temporal monitoring employed in this study over 15 successive months, and the distinction regarding the condition of the stranded clams (dying or dead), allowed an unprecedented understanding of the frequency, magnitude and factors affecting the mortality events of beach clams, using *T*. *mactroides* in Caraguatatuba Bay as a study case.

The overall characterization of the dying and dead stranded individuals strongly supported the interpretation that stranded individuals derive from the intertidal/shallow-subtidal population stratum. This evidence includes the simultaneous presence of dying and dead stranded individuals in the upper intertidal zone, and that most of the dead individuals had their valves closed. In addition, the stranded individuals belonged to the same cohort as the buried intertidal individuals, given the close relationship of their size structure, since individuals from subtidal areas are on average much smaller than those from the intertidal zone [33]. This result highlights the ecological and behavioral components of this process, such as intertidal thermal stress, the swash dynamic, and the implications of passive displacement and relocation in this environment.

Distinguishing between dying and dead individuals showed that there is a significant relationship between the two categories. The density of dying individuals corresponded better to environmental factors, probably because they die over a short time period, which doubtless presents pseudoreplication by the sampling method used. However, separation by size structure must be done carefully. The mean dimensions of shells of dying clams were consistently larger than those of dead clams, reflecting the greater ability of larger individuals to tolerate exposure to air, since they are more resistant to desiccation [40]. Therefore, working only with dying clams may introduce biases into the size-structure information.

The temporal patterns indicated that stranding of *T*. *mactroides* in Caraguatatuba Bay occurs frequently but irregularly. Even though the intensity of these events decreased considerably near the end of the study, stranding occurred in nearly the entire monitoring period, with large numbers of stranded individuals appearing sporadically. Mean daily numbers of stranded clams during October and November 2007 reached an impressive ~150 ind.m^-2^. The stranding events were widely distributed along this 4-km stretch of the bay, as indicated by the low standard errors of most density values.

Although quantitative data for mass-mortality events of sandy-beach species are practically nonexistent, the mean density of stranded individuals of *T*. *mactroides*, 27.09 ind.m^-2^, seems high. This is particularly true considering the frequency and spatial extent of these events in Caraguatatuba Bay during the study period, but also comparing these values to estimates for the *T*. *mactroides* population in this same bay during a previous period (2003–2004), when the population density was about 14 ind.m^-2^ [33]. The overall mean density of stranded individuals of *T*. *mactroides* was higher than densities recorded for *Tivela isabelleana* (4–15 ind.m^-2^) during mortality events, but lower than recorded for the yellow clam *Mesodesma mactroides* (over 60 ind.m^-2^), both on Argentinean beaches [3,13]. The broad across- and along-shore distribution of *T*. *mactroides* in the extensive Caraguatatuba Bay (~16 km long) indicates that a large amount of carrion derived from the stranded individuals becomes available to the beach-surf/swash system and the adjacent terrestrial environment, since these clams are eaten by several marine species and the stranded individuals are also likely to attract a wide range of scavengers to the beach [20,32,50].

Burrowing clams need a wet substrate to allow the foot to penetrate and anchor before pulling the shell down, and also for the shell to remain in the same location until burrowing is completed, avoiding rolling by waves (*sensu* [39]). This situation is more critical for *T*. *mactroides*, which in contrast to some bivalve species that burrow quickly (e.g. *Donax*) [40], needs considerable time to bury and escape wave action. When burrowing in the upper swash zone of Caraguatatuba Bay, *T*. *mactroides* constricts the valves, squirts water out every 4 seconds, and buries completely only after about 20 to 30 squirts (A. Turra, pers. observation). Considering the difficulty of burying as well as the effect of low-energy waves in adding sediment to the beach [40], presumably, individuals digging out during low-energy events are more likely to be carried higher up on the beach. The surviving clams that reach the upper beach zone are then unable to return to the intertidal wet zone, because of the low wave energy that reaches that portion of the beach (weak backwash). In addition, the dry compact sand in the upper intertidal zone precludes burrowing and survival of these stranded individuals. In short, clams that are unable to bury deep enough in time would be washed up to the upper intertidal zone, by the action of an accretive wave regime, where they strand and desiccate.

This process accounts well for the negative relationship between mortality and wave height recorded here: smaller waves favor the deposition of individuals higher on the beach. Meanwhile, the higher the wave the farther from the beach it breaks, and the stronger the backwash [51], which may combine to prevent these burying intertidal individuals from being stranded. The negative effect from the harsher swash climate toward reflective conditions (lower waves), as postulated by the Habitat Harshness Hypothesis [52,53], has been widely documented for intertidal macrofauna, as also observed for this *T*. *mactroides* population, which is less dense in reflective than in dissipative conditions [33]. The negative relationship between mortality and wave height identified here is notable because stranding of mollusks, mainly bivalves, is commonly attributed to storms [3,6,18,40]. In fact, extreme conditions are prone to result in high mortality of a variety of organisms, but the present observations show that as a rule, higher mortality events were actually associated with previous low-wave-energy events. This highlights the importance of studies on finer time scales to understand certain important ecological relationships, especially in dynamic environments, and the need for these assessments in order to improve understanding and management of coastal resources.

There was no significant relationship between mortality intensity and population density, but the density of the living population will, almost as a matter of course, influence the density of stranded individuals. Data on the living population were not available with the same periodicity as the stranding data, and this lack may have resulted in the failure to identify this effect. Still there was some evidence of this relationship, most clearly in the sharp decrease in the density of stranded individuals toward the end of the study period, which was associated with a decrease in density of the living population.

For the same species and area, Turra et al. [32] found a similar natural mortality index (Z) for periods of very different densities, and showed that density-dependent processes were important in governing population dynamics. However, with respect to stranding density, the largest stranding events coincided with high population densities. The analysis of the size structure supported this suggestion, showing that the size of stranded individuals primarily reflected the size structure of the intertidal population stratum. However, whatever the population density, other factors will determine whether and which fraction of the population will die. For example, in the present study, the three cases of highest mortality (Fig 3) occurred in October and November 2007, and so may have been related to a period of high population densities. Nevertheless, the lowest mortality events were related to greater wave heights, even when the population density was quite high. Thus, the influence of wave height was very consistent, and it is reasonable to hypothesize that varying population densities would result, in the quantile regression analysis (or “envelope”), in different values for the intercept and slope of the relationship between wave height and stranding density.

Many other factors may be related to mass mortality in mollusks. A fairly well-recognized factor is the effect of parasitism, which may be of high or low magnitude, causing the death of a small portion or the collapse of a population [10,13,54]. Fiori et al. [13] suggested that parasitism by a protozoan may have caused the mass-mortality events of the yellow clam *Mesodesma mactroides* along its entire distribution range. This hypothesis was based on a mortality event in an Argentinean yellow-clam population infested by a coccid protozoan, which caused necrosis in the gills and stomach of some infested individuals (21.4%). Denadai et al. [55] observed parasitism in gonads of *T*. *mactroides* in Caraguatatuba Bay in the 2003–2004 period. Trematodes were found in about 10% of the individuals, and caused total infertility in some of them. In contrast to the episodic mortality events that dramatically decrease populations of *M*. *mactroides* [13], the mortality events of *T*. *mactroides* were more continuous in Caraguatatuba Bay. Thus, given the hydrodynamic and population-density aspects observed here, parasitism must not have been a main factor. However, a more-specific evaluation of parasites and pathological bacteria in this population in future mortality events is recommended, since it is a recognizable factor affecting mortality. Also, over a wider spatial scale there might be other environmental factors, such as beach slope and sediment texture, that may influence the stranding and mass mortality of *T*. *mactroides*, and could be important to understand and manage these events.

It is important to understand these high-mortality events, since large numbers of stranded clams may cause socioeconomic and environmental problems for the local residents. Harvesting of this species decreased by one-third during high-mortality periods [34]. The strong unpleasant odor of the dead clams accumulated on the sand affects tourism and raises public-health concerns, because the clams are decomposing along the most intensively used part of the beach. Strategies to utilize this very high biomass as a solution for this environmental and socioeconomic problem should be discussed and implemented. The use of meat and shells in animal feeds for aquaculture, biodiesel production from animal fat, and use of calcium carbonate for agriculture or as an aquarium substrate are some alternatives. Nevertheless, it is necessary to undertake long-term monitoring of some of these populations to better comprehend, and predict, the dynamics of density variation.

In summary, stranding and mass-mortality events of *T*. *mactroides* were frequent and widespread along the extensive beaches of Caraguatatuba Bay in 2007–2008. The size structure of the stranded clams supports the supposition that the death of stranded individuals occurs in the intertidal and shallow subtidal zone, highlighting the importance of ecological and behavioral components to mortality processes. The wave height three days before the samplings explained much of the variation in mortality events of *T*. *mactroides*; the higher the waves, the smaller the number of stranded individuals. This is likely due to the accretive nature of low-energy waves, so that individuals in the process of digging out would be easily carried upward, while higher waves break seaward and have a stronger backwash, preventing the clams from stranding on the uppermost part of the beach. This is an important finding for the ecology of sandy-beach bivalves, and future, temporally refined studies should examine this phenomenon further. Similarly, long-term studies on the mortality and living population of clams, concurrently with environmental features, are important for appropriate management of this resource.

## References

[1] Sherman BH. Marine ecosystem health as an expression of morbidity, mortality and disease events. Mar Pollut Bull. 2000;41: 232–254.

[2] Gonzalez SA, Stotz WB, Aguilar M. Stranding of scallops related to epiphytic seaweeds on the coast of northern Chile. J Shellfish Res. 2001;20: 85–88.

[3] López RA, Penchaszadeh PE, Marcomini SC. Storm-related strandings of mollusks on the northeast coast of Buenos Aires, Argentina. J Coast Res. 2008;24: 925–935.

[4] Olivier SR, Capezzani DAA, Carreto JI, Christiansen HE, Aizpun de Moreno JE, Penchaszadeh PE. Estructura de la comunidad, dinámica de la población y biología de la almeja amarilla (*Mesodesma mactroides* Desh. 1854) en Mar Azul (Pdo. de Gral Madariaga, Bs. As., Argentina). Proy Desarr Pesq FAO, Ser Inf Técn. 1971;27: 1–90.

[5] Arntz WE, Valdivia E. Incidencia del fenómeno “El Niño” sobre los mariscos en el litoral peruano Simposio “El Niño”; compilado en Boletín (volumen extraordinario) IMARPE: “El Niño” Su impacto en la fauna marina. Callao, Peru: Instituto de mar del Peru; 1985 pp. 91–101.

[6] Rees HL, Dare PJ. Sources of mortality and associated life-cycle traits of selected benthic species: a review. Maff Direct Fish Res. 1993;33: 1–36.

[7] Odebrecht С, Rörig L, Garcia V, Abreu P. Shellfish mortality and a red tide event in southern Brazil In: Lassus P, Arzul G, Erard E, Gentien P, Marcaillou C, editors. Harmful marine algal blooms. Paris: Lavoisier Publishing; 1995 pp. 213–218.

[8] Dyrynda E a., Law RJ, Pipe RK, Ratcliffe N a. F8 4:00 Modulations in cell-mediated immunity of mussels (*Mytilus edulis*) following the “sea empress” oil spill. Dev Comp Immunol. 1997;21: 124 10.1016/S0145-305X(97)88577-1

[9] Riascos JM, Carstensen D, Laudien J, Arntz WE, Oliva ME, Guntner A, et al Thriving and declining: Climate variability shaping life-history and population persistence of *Mesodesma donacium* in the Humboldt Upwelling System. Mar Ecol Prog Ser. 2009;385: 151–163. 10.3354/meps08042

[10] Riascos JM, Heilmayer O, Oliva ME, Laudien J, Wolf a. Infestation of the surf clam Mesodesma donacium by the spionid polychaete Polydora bioccipitalis. J Sea Res. 2008;59: 217–227. 10.1016/j.seares.2008.01.003

[11] Ortega L, Castilla JC, Espino M, Yamashiro C, Defeo O. Effects of fishing, market price, and climate on two South American clam species. Mar Ecol Prog Ser. 2012;469: 71–85. 10.3354/meps10016

[12] Aburto J, Stotz W. Learning about TURFs and natural variability: Failure of surf clam management in Chile. Ocean Coast Manag. 2013;71: 88–98. 10.1016/j.ocecoaman.2012.10.013

[13] Fiori S, Vidal-Martinez VM, Sima-Alvarez R, Rodriguez-Canul R, Aguirre-Macedo ML, Defeo O. Field and laboratory observations of the mass mortality of the yellow clam *Mesodesma mactroides* in South America: the case of Isla del Jabalí, Argentina. J Shellfish Res. 2004;23: 451–455.

[14] Johnson PT. Population crashes in the bean clam, *Donax gouldi*, and their significance to the study of mass mortality in other marine invertebrates. J Invertebr Pathol. 1968;12: 349–358. 5752488 10.1016/0022-2011(68)90339-x

[15] Fiori SM, Cazzaniga NJ. Mass mortality of the yellow clam, *Mesodesma mactroides* (Bivalvia: Mactracea) in Monte Hermoso beach, Argentina. Biol Conserv. 1999;89: 305–309. 10.1016/S0006-3207(98)00151-7

[16] Greenfield DI, Lonsdale DJ. Mortality and growth of juvenile hard clams *Mercenaria mercenaria* during brown tide. Mar Biol. 2002;141: 1045–1050. 10.1007/s00227-002-0890-x

[17] Riascos JM, Heilmayer O, Oliva ME, Laudien J. Environmental stress and parasitism as drivers of population dynamics of *Mesodesma donacium* at its northern biogeographic range. ICES J Mar Sci. 2011;68: 823–833. 10.1093/icesjms/fsr026

[18] McLachlan A, Dugan JE, Defeo O, Ansell AD, Hubbard DM, Jaramillo E, et al Beach clam fisheries. Oceanogr Mar Biol an Annu Rev. 1996;34: 163–232.

[19] Dadon JR. Changes in the intertidal community structure after a mass mortality event in sandy beaches of Argentina. Contrib to Zool. 2005;74: 27–39. Available: http://dpc.uba.uva.nl/ctz/vol74/nr01/art02

[20] Turra A, Fernandez, Wellington S Bessa E, Santos FB, Denadai MR. Multi-species generalist predation on the stochastic harvested clam *Tivela mactroides* (Mollusca, Bivalvia). Estuar Coast Shelf Sci. 2015;

[21] Arntz WE, Brey T, Tarazona J, Robles a. Changes in the structure of a shallow sandy-beach community in Peru during an El Niño event. South African J Mar Sci. 1987;5: 645–658. 10.2989/025776187784522504

[22] Defeo O. Marine invertebrate fisheries in sandy beaches: an overview. J Coast Res. 2003;35: 56–65.

[23] Thiel M, Macaya EC, Acuna E, Arntz WE, Bastias H, Brokordt K, et al The Humboldt Current System of northern and central Chile: oceanographic processes, ecological interactions and socioeconomic feedback. Oceanogr Mar Biol. 2007;45: 195–344.

[24] Defeo O., De Alava A., Valdivieso V., & Castilla JC. Historical landings and management options for the genus *Mesodesma* in coasts of South America. Biol Pesq. 1993;22: 41–54.

[25] Castilla JC, Defeo O. Latin American benthic shellfisheries: Emphasis on co-manageent and experimental practices. Rev Fish Biol Fish. 2001;11: 1–30. 10.1023/A:1014235924952

[26] Defeo O, Castrejón M, Ortega L, Kuhn AM, Gutiérrez NL, Castilla JC. Impacts of Climate Variability on Latin American Small-scale Fisheries. Ecol an. 2013;18: 30 10.5751/ES-05971-180430

[27] Turra A, Xavier LY, Pombo M, de Carvalho Paschoal C, Denadai MR. Assessment of recreational harvesting of the trigonal clam *Tivela mactroides*: Socioeconomic aspects and environmental perception. Fish Res. 2016;174: 58–67. 10.1016/j.fishres.2015.08.026

[28] Tata A, Prieto A. Producción secundaria en una población del bivalvo tropical *Tivela mactroides* (Veneridae) en el Oriente de Venezuela. Caribb J Sci. 1991;21: 63–73.

[29] Mendoza JJ, Marcano JS. Abundancia y evaluación del guacuco *Tivela mactroides* en la Ensenada La Guardia, Isla de Margarita, Venezuela. Boletín del Inst Ocean Venez. 2000;39: 79–91.

[30] Arrieche D, Prieto A. Population parameters of the trigonal tivela *Tivela mactroides* (Bivalvia: Veneridae) from Caicara Beach, Anzoátegui, Venezuela. Ciencias 3 2006;32: 285–296.

[31] Crescini R, Villalba W, Prieto A, Leiva R. Parametros de crecimiento y mortalidad de *Tivela mactroides* (Veneridae) en la Enseada La Guardia, Isla de Margarita, Venezuela. Bol del Inst Oceanogr Venez. 2012;51: 85–91.

[32] Turra A, Petracco M, Amaral ACZ, Denadai MR. Temporal variation in life-history traits of the clam *Tivela mactroides* (Bivalvia: Veneridae): Density-dependent processes in sandy beaches. Estuar Coast Shelf Sci. Elsevier Ltd; 2014;150: 157–164. 10.1016/j.ecss.2013.06.004

[33] Turra A, Petracco M, Amaral ACZ, Denadai MR. Population biology and secondary production of the harvested clam *Tivela mactroides* (Born, 1778) (Bivalvia, Veneridae) in Southeastern Brazil. Mar Ecol. 2014; 1–14. 10.1111/maec.12137

[34] Denadai MR, Pombo M, Bernadochi LC, Turra A. Harvesting the Beach Clam *Tivela mactroides*: Short- and Long-Term Dynamics. Mar Coast Fish. 2015;7: 103–115. 10.1080/19425120.2015.1007183

[35] Rios EC. Seashells of Brazil. Rio Grande, Brazil: Museu Oceanográfico da Fundaçao Universidade do Rio Grande; 1985.

[36] Narchi W. Comparative study of the functional morphology of *Anomalocardia brasiliana* (Gmelin, 1791) and Tivela mactroides (Born, 1778)(Bivalvia, Veneridae). Bull Mar Sci. 1972;22: 643–670.

[37] Denadai MR, Cecília Z. Amaral A., Turra A. Along‐ and across‐shore components of the spatial distribution of the clam *Tivela mactroides* (Born, 1778) (Bivalvia, Veneridae). J Nat Hist. 2005;39: 3275–3295. 10.1080/00222930500126156

[38] Herrera A. BD. Influence of riverine outputs on sandy beaches of Higuerote, central coast of Venezuela. Lat Am J Aquiatic Res. 2011;39: 56–70. 10.3856/vol39-issue1-fulltext-6

[39] Trueman ER, Brand a R, Davis P. The dynamics of burrowing of some common littoral bivalves. J Exp Mar Bio Ecol. 1966;44: 469–492.

[40] McLachlan A, Brown AC. The ecology of sandy shores Academic Press; 2010.

[41] Tolman HL. User manual and system documentation of WAVEWATCH-III version 2.22 Washington, USA: NOAA/NWS/NCEP/MMAB; 2002 p. 133.

[42] Holthuijsen LH, Booij N, Ris RC. A spectral wave model for the coastal zone. 2nd International Symposium on Ocean Wave Measurement and Analysis. 1993. pp. 630–641.

[43] Zar JH. Biostatistical analysis New Jersey: Pearson Prentice Hall; 2010.

[44] Zuur A, Ieno EN, Walker N, Saveliev AA, Smith GM. Mixed effects models and extensions in ecology with R. 2009.

[45] Pinheiro J, Bates D, DebRoy S, Sarkar D, Team RC. nlme: Linear and Nonlinear Mixed Effects Models. R package version 3.1–120. 2015.

[46] Cade BS, Noon BR. A gentle introduction to quantile regression for ecologists. Front Ecol Environ. 2003;1: 412–420. 10.1890/1540-9295(2003)001[0412:AGITQR]2.0.CO;2

[47] Caddy JF, Defeo O. Enhancing or restoring the productivity of natural populations of shellfish and other marine invertebrate resources Food and Agriculture Organization of the United Nations; 2003.

[48] Austin MP. Species distribution models and ecological theory: A critical assessment and some possible new approaches. Ecol Modell. 2007;200: 1–19. 10.1016/j.ecolmodel.2006.07.005

[49] Wood S. Generalized additive models: an introduction with R CRC press; 2006.

[50] Schlacher T, Strydom S, Connolly R, Schoeman D. Donor-Control of Scavenging Food Webs at the Land-Ocean Interface. PLoS One. 2013;8: e68221 23826379 10.1371/journal.pone.0068221 PMC3694906

[51] Masselink G, Puleo J A. Swash-zone morphodynamics. Cont Shelf Res. 2006;26: 661–680. 10.1016/j.csr.2006.01.015

[52] Defeo O, Gomez J, Lercari D. Testing the swash exclusion hypothesis in sandy beach populations: the mole crab *Emerita brasiliensis* in Uruguay. Mar Ecol Prog Ser. 2001;212: 159–170. Available: http://www.int-res.com/abstracts/meps/v212/p159-170/

[53] Defeo O, Martínez G. The habitat harshness hypothesis revisited: life history of the isopod *Excirolana braziliensis* in sandy beaches with contrasting morphodynamics. J Mar Biol Assoc United Kingdom. 2003;83: 331–340.

[54] Penn D, Jane Brockmann H. Age-biased stranding and righting in male horseshoe crabs, *Limulus polyphemus*. Anim Behav. 1995;49: 1531–1539. 10.1016/0003-3472(95)90074-8

[55] Denadai MR, Le Sueur-Maluf L, Marques CG, Amaral AC, Adamo I, Yokoyama LQ, et al Reproductive cycle of the trigonal clam *Tivela mactroides* (Bivalvia, Veneridae) in Caraguatatuba Bay, southeastern Brazil. Mar Biol Res. 2015;11: 847–858.

